# Methodological optimization for efficient degradation of Acid Violet 49 using advanced oxidation processes and varied photocatalyst combinations

**DOI:** 10.1038/s41598-024-81409-8

**Published:** 2024-12-05

**Authors:** Aqsa Iqbal, Tanveer Hussain Bokhari, Muhammad Usman, Amnah Yusaf, Asim Mansha, Muhammad Saeed, Salahuddin Khan, Mazhar Iqbal, Muhammad Ahsan Bhatti, Syed Salman Shafqat, Noshin Afshan, Muhammad Nadeem Zafar

**Affiliations:** 1 Colloidal and Computational Lab, Department of Chemistry, Government College University Faisalabad, Faisalabad, 38000 Pakistan; 2 College of Engineering, King Saud University, P.O. Box 800, Riyadh, 11421 Saudi Arabia; 3 National Institute for Biotechnology and Genetics Engineering (NIBGE), Faisalabad, 38000 Pakistan; 4 Department of Zoology, Riphah International University, Faisalabad, 38000 Pakistan; 5 Department of Chemistry, Division of Science and Technology, University of Education, Lahore, 54770 Pakistan; 6 School of Life Sciences, Shandong Cancer Hospital and Institute, Shandong First Medical University & Shandong Academy of Medical Sciences, Jinan, 250117 China; 7 Department of Chemistry, University of Gujrat, Gujrat, 50700 Pakistan

**Keywords:** Photodegradation, H_2_O_2_, TiO_2_, ZnO, SnO_2_, LC-MS, FTIR, Computational biology and bioinformatics, Chemistry, Materials science, Nanoscience and technology

## Abstract

This study investigates the photodegradation of Acid Violet 49 in an aqueous medium under UV, UV/H_2_O_2,_ and combined with photo catalyst (UV/H_2_O_2_/TiO_2_, UV/H_2_O_2_/ZnO, and UV/H_2_O_2_/SnO_2_). The impact of all operational parameters including catalytic dosage, peroxidation (H_2_O_2_), pH and dye concentration were evaluated. The degradation efficiency of AV49 was enhanced up to 96% with the alternative photocatalyst UV/H_2_O_2_/ZnO under the circumstances of 0.6mL H_2_O_2_, 0.9 g ZnO, 50 mg/L initial dye concentration, and pH 9 after 90 min. Hemolytic test were used to check the toxicity level of products. FTIR spectroscopy of UV/ZnO/H_2_O_2_ was applied to identify the functional group of AV49 before and end product of the degradation. Moreover, the analytical technique LC-MS provided valuable information regarding the degradation products of AV 49. A proposed pathway was identified based on the findings of the degradation product.

## Introduction

Dyes are a hotly debated problem when dealing with organic effluents because they are chemically as well as photolytically stable. They are designed to resist the elements of light, water, weather, and detergents^1^. Dyes are defined as organic aromatic compounds that absorb light at the wavelength range of 400–800 nm (visible spectrum)^2^. Almost 10 to 15% of dyes are released during the dying process. Bright-colored dyes, such as reactive and acid dyes, are water-soluble and hence the most difficult to degrade. Synthetic dyes are widely utilized in the dying process and are the main pollutants found in industrial wastewater^3^. According to the World Dye Consumption Report 2017 belonging to IHS Markit, Asian countries consume 75% of all synthetic dyes manufactured. Every year almost 140,000 tons of synthetic dyes are released in aqueous streams into the environment^4^. According to the US Department of Commerce, textile manufacturing of dyes increased 3.5 folds between 1975 and 2020^5^.

Traditional processes for the degradation of dyes cannot adequately handle the enormous chemical complexity of dyes. Moreover, these technologies are causing new problems, such as increased sludge formation, which need further treatment. Over the last few decades, advanced oxidation processes (AOPs) have proven to be an effective and successful method of treatment for decomposing refractory chemicals and degradation of contaminants that are harmful, inhibiting, or recalcitrant^2,5^.

Advanced oxidation processes (AOPs) are used for the generation of free active hydroxyl radicals (^**·**^OH) as a primary oxidant and are becoming a more realistic choice for dealing with industrial effluents^5^. AOPs based on the production of reactive hydroxyl radicals (^**·**^OH), such as UV, UV/H_2_O_2_, and UV/ H_2_O_2_/TiO_2_, ZnO, and SnO_2_ -mediated photocatalysis processes, have the emerged potential for degradation of an aqueous solution of dye^6^. The majority of organic compounds in industrial effluents can be oxidized and even mineralized by hydroxyl radicals^7^. The hydroxyl radical has a 2.78 eV oxidation potential, which allows it to eliminate contaminants in wastewater. As a result, one of the AOPs that has been successfully employed in the degradation of dye is a combined UV and hydrogen peroxide oxidation method^8^. One major benefit of AOPs is their capacity to fully degradation organic materials into CO_2_ and H_2_O. There may be no waste sludge generation after full mineralization, depending on the processes, features, and kinds of AOPs. Target compounds in intricate combinations that have large band gaps have been targeted by AOPs using a single technique. Additionally, AOPs have excellent treatment efficacy for varying quantities of organic compounds, such as dye, and can destroy a broad variety of dissolved organic materials (DOM). The running cost of modern oxidation technologies is the most noticeable disadvantage when compared to other traditional physicochemical or biological treatments^9^.

Furthermore, the UV/H_2_O_2_ process has significant advantages over conventional AOPs. (i) During the treatment process, no sludge forms. (ii) It can be done in normal settings. (iii) This process produces oxygen, which is useful for aerobic biological decomposition^10^. Acid violet 49 is widely used bright blue purple (violet powder) dye. It dissolves well in cold and hot water and is soluble in ethanol. When heated to decompose AV 49 produces toxic fumes of nitrogen oxide, ammonia, sodium oxide, and sulfur oxides^11^. The UV from the reactor attacks the O-O bond in H_2_O_2_ during the mixing phase, allowing the formation of hydroxyl radicals. As seen in the following reaction, the UV/H_2_O_2_ combination makes it easier for UV to split H_2_O_2_ and form hydroxyl radical^12^.

$$\:\:\:\:\:\:{H}_{2}{O}_{2\:}$$+ $$\:hv$$ → $$\:2{HO}^{.}$$

$$\:{H}_{2}{O}_{2}$$ + $$\:H{O}^{.}$$ → $$\:H{O}_{2}$$. + $$\:{H}_{2}$$O

$$\:{\:H}_{2}{O}_{2}$$+ $$\:{HO}_{2}$$. → $$\:{HO}^{.}$$ + $$\:{H}_{2}$$O + $$\:{O}_{2}$$

$$\:2{HO}^{.}$$ → $$\:{H}_{2}{O}_{2}$$

$$\:{\:2HO}_{2}$$. → $$\:{H}_{2}{O}_{2}$$ + $$\:{O}_{2}$$

$$\:\:\:{HO}^{.}$$+ $$\:{HO}_{2}$$. → $$\:{H}_{2}O$$ + $$\:{O}_{2}$$

Heterogeneous photocatalysis with TiO_2_, SnO_2_, and ZnO nanoparticles is thought to be a viable approach for converting harmful compounds and dyes to harmless species (CO_2_, H_2_O, etc.). Nanostructured TiO_2_ has had a wide range of uses since the advent of nanotechnology^13^. Photocatalytic degradation is an advanced oxidation process in which a semiconductor catalyst is exposed to UV light to produce hydroxyl radicals. UV light energy excites valence electrons and allows electron transfer from the valence band (VB) to the conduction band (CB) by irradiating the catalyst surface. As a result of electron transfer, electron-hole pairs are formed. When electron-hole pairs react with hydroxide ions or water molecules hydroxyl radicals (^**·**^OH) are formed^14^. A semiconductor (such as TiO_2_, ZnO, Al_2_O_3_, RuO_2_, SiO_2_, WO_3_, SnO_2_, ZrO_2_, CdS, etc.) and UV light are used in photocatalytic systems.

Among all catalysts ZnO, TiO_2_, and SnO_2_ are the most efficient and cost-effective. SnO_2_ photocatalysis of dyes is rarely reported, due to its mixed phases of SnO_2_ and ZnO. Titanium dioxide (TiO_2_) is thought to be a more effective catalyst for the photocatalytic degradation of dye because of its higher stability in an aqueous solution and its excellent bandgap^15^. TiO_2_ shows a weak photoresponse to the visible light region, as well as a high electron (e^−^) hole (h^+^) pairs recombination rate greatly reducing the quantum efficiency, both of which limited the further widespread application of TiO_2_ for solar energy conversion, environmental cleanup and hydrogen generation^16^. The encountered issue with TiO_2_ is that it has less separability from the reaction mixture. ZnO powder has a better quantum efficiency than TiO_2_, ZnO is a viable replacement for TiO_2_ due to its larger surface area. ZnO has well-efficient photocatalytic activities belonging to the n-type semiconductor^17^. It absorbs a large amount of visible light due to its property of strong luminescence. In comparison to TiO_2_ (E_bg_ = 3.2 eV), zinc oxide has a wide bandgap (E_bg_ =3.27 eV) and produces large amounts of hydroxyl radicals^18^. Due to its exceptional catalytic, optical, and electrical qualities as well as its numerous potentials uses in the environmental and energy sectors, zinc oxide, or ZnO, is also one of the materials that has been studied the most^17,18^. The degradation potential of these catalysts is significantly influenced by other physical variables, including surface area and zeta potential, which basically evaluates how stable a system is. Stronger repulsive forces between catalyst particles result from higher zeta potential values, providing them with more stability. In photocatalytic processes, catalysts are used in suspension and stable forms^19^.

A recent study of this article reveals the effectiveness of UV, UV/H_2_O_2_, UV/H_2_O_2_/TiO_2_, UV/H_2_O_2_/ZnO, and UV/H_2_O_2_/SnO_2_ processes as an advanced oxidation process for the degradation of Acid Violet 49 from aqueous solutions. The impact of several operational parameters, including dye concentration, commercially available catalysts, H_2_O_2_, and various pH scales, were studied in ongoing research. The catalyst and the light source are the most significant and fundamental parts for the successful and complete mineralization.

## Materials and methods

### Model compound and chemicals

**Fig. 1:**
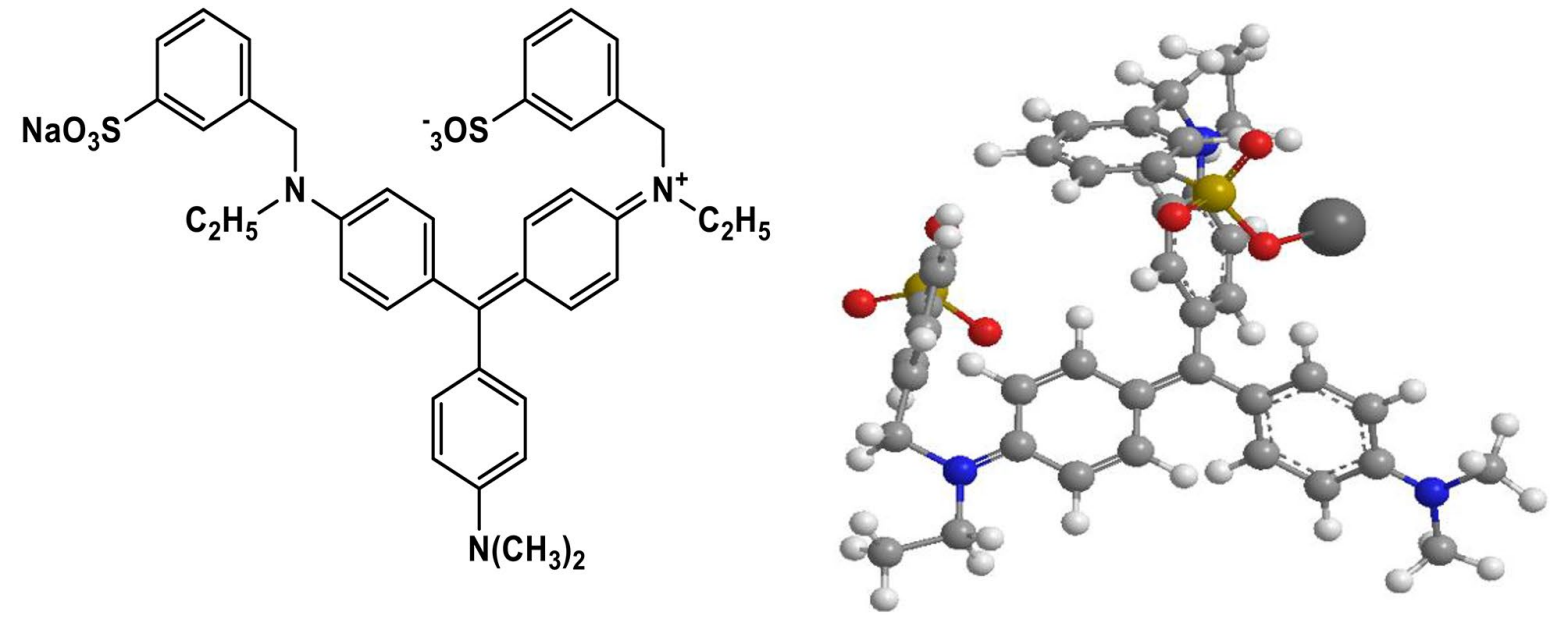
Chemical Structure of Acid Violet 49.

AV49 dye has a molecular weight of 733.89 g/mol and the chemical formula C_39_H_41_N_3_NaO_6_S_2_ was obtained from Dystar Pakistan (Pvt) Ltd Karachi. Its structure is given in Fig. 1. The catalysts ZnO, TiO_2_, SnO_2_ (99%), hydrogen peroxide (30% V/V, density 1.11 g/mL) and NaOH and HCl (99%) were purchased from Sigma-Aldrich. The aqueous solution of AV 49 was used as a compound of interest for the study. Distilled water was used throughout the experiment. The general properties of Acid Violet 49 can be seen in Table 1.

**Table 1:** General properties of Acid Violet 49.

Acid violet 49	Properties
Molecular formula	C_39_H_40_N_3_NaO_6_S_2_
Molecular weight	733.89 g/mol
C.I number	42,640
CAS number	1694-09-3
Chromophore	P-quinoid group

### Reactor set-up

The photocatalytic oxidation processes were carried out in a photochemical reactor with eight UV lamps within a chamber. According to the information which is taken from the manufacturer, each lamp produces 254 nm light with a 144-watt intensity for 90% of its output. The sample and UV irradiation source, which were employed combined to create hydroxyl radicals, were separated by about 2 cm.

### Operational conditions

A stock solution of the target compound Acid Violet 49 (AV49) was prepared with a specific concentration (1,000 mg/L) and placed in the dark. A 100mL of AV49 solutions with specific varying concentrations of different parameters were put into the UV reactor to analyze the effects of different dye concentrations (50, 100, and 150 mg/L), different pH (3, 5, 7, and 9), different catalysts TiO_2_, SnO_2_, or ZnO (0.3,0.6, and 0.9 g), and different times on the degradation of acid violet 49. The pH of the operating samples was pre-adjusted by applying 0.1 M HCl and NaOH. The UV lamp was switched on for at least 30 min before the experiment to guarantee a constant light output. The operating sample was covered with aluminum foil during irradiation to prevent light exposure and any unwanted side effects.

### UV/H2O2 process

The different concentrations of AV 49 solution with hydrogen peroxide ranging from 0.2 to 0.6mL were put into the photoreactor and irradiated for the specified time. The dye absorption spectra were recorded when the sample was removed at the end of the exposure. A double-beam spectrophotometer was used to measure the test solution against a blank.

### UV/ Nano-catalyst process

**Fig. 2:**
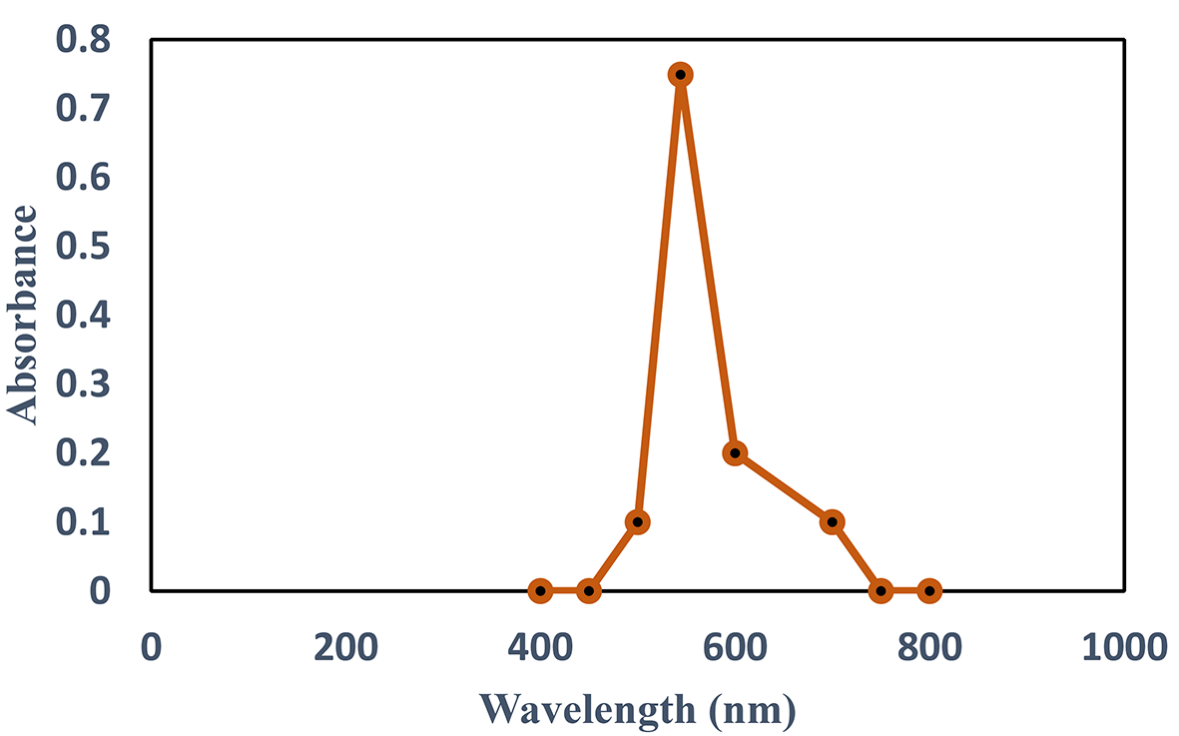
Absorption band for AV49.

An aqueous solution of AV49 with a known quantity of catalytic powder was placed in the reactor. Before irradiation, the suspensions were magnetically stirred in the dark for 30 min to confirm that the dye and the catalytic surface were in a state of adsorption/desorption equilibrium. Samples were obtained at predetermined reaction times and then filtered with 0.22 μm filters to eliminate catalyst particles. According to Beer’s Law, the dye concentration was calculated using a UV-Visible spectrophotometer at the highest absorption wavelength. The maximum absorption wavelength of the AV49 solution (λ_max_ = 544 nm) in (Fig. 2) was measured using HITACHI UH5-300 double beam spectrophotometer. All catalysts were subjected to the same technique.

### Analytical method

The prominent peak for AV 49 was observed at λ_max_ 544 nm. The reduction in maximum absorption at 544 nm was used to calculate the degree of degradation.

$$Percentage{\rm{ }}\left( \% \right){\rm{deg}}radation\, = \:\frac{{\:\:Co\:\: - \:\:\:{C_t}}}{{\:{C_t}}}\: \times \:100$$

where C_o_ is the absorbance of AV 49 solution before irradiation and C_t_ is the absorbance after irradiation.

### LC-MS/FTIR

LC-MS was used to identify the degraded product of AV49 in the aqueous phase and performed at the National Institute of Biotechnology and Genetic Engineering (NIBGE) Faisalabad. The reaction intermediates were examined by Electrospray Ionization (ESI-LC/MS) equipped with a Linear Ion Trap Mass spectrometer (ITMS).

To identify the functional group of AV49 before and after the degradation FTIR was done at the University of Agriculture Faisalabad. The aqueous irradiation samples were extracted with acetone for FTIR analysis. The organic phase was concentrated and FTIR analysis was performed on it.

## Results and discussion

### UV-Visible Spectra

**Fig. 3:**
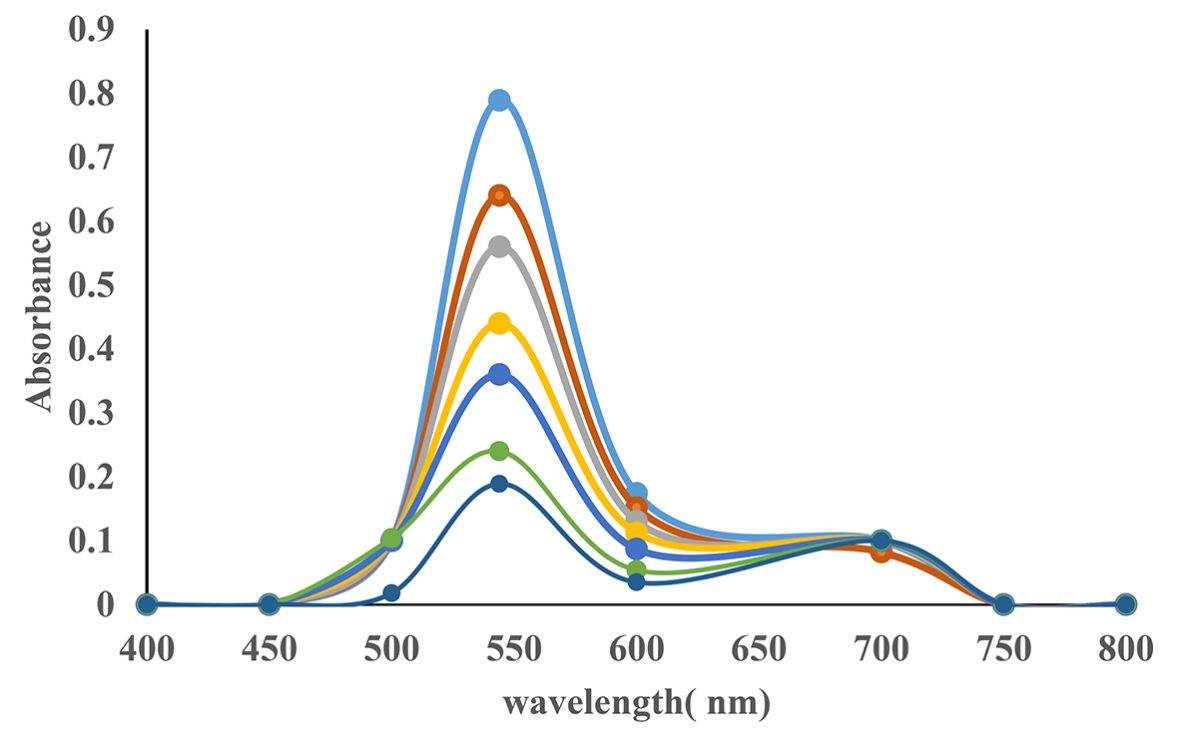
Absorption spectra of Acid Violet 49 at various irradiation times.

The spectrum for the dye AV49 was obtained using the ultraviolet/visible (UV/Vis) spectrophotometry technique following spectral scanning from 200 to 800 nm. The notable wavelengths (λ) of 250 and 300 nm (auxochromes), and 544 nm (chromophore groups) were determined. It can be seen in Fig. 3 that the percentage of degradation was enhanced gradually with increasing time. The percentage of degradation for 50ppm was 18% at 0 min. When the time was 45 min to 90 min after radiation the percentage of degradation increased from 44 to 77%.

### Effects of AV49 concentration

**Fig. 4:**
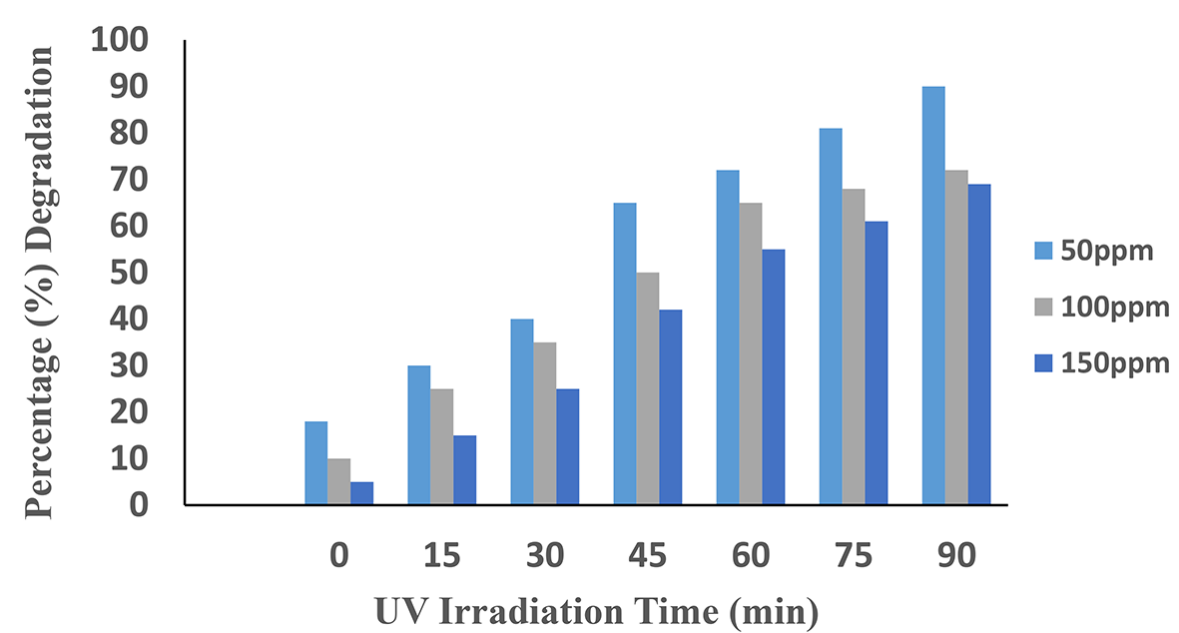
Effects of concentration of dye on degradation efficiency of AV49.

The effect of this parameter on degradation was examined by varying the concentration throughout a range of 50 ppm to 150 ppm. It can be seen from (Fig. 4) that by increasing the dye concentration from 50 to 150 mg/L, the degradation efficiency decreased from 90 to 69%, at a contact time of 90 min as shown in Table 2. It is due to the limited rate of production of hydroxyl radicals. So, at a constant time, the rate of radical production will be the same by increasing the concentration of dye, the number of produced radicals will not be enough for the complete mineralization of dye^20^.

**Table 2:** Degradation of the target compound Acid Violet 49 in the UV, UV/H_2_O_2_, UV/H_2_O_2_/ZnO, UV/H_2_O_2_/ TiO_2_, and UV/H_2_O_2_/SnO_2_ processes.

AOPs	Radiation	Parameters				Percentage (%) Degradation		
UV	254 nm	Time (min)				50ppm	100ppm	150ppm
		0				18	10	5
		15				30	25	15
		30				40	35	25
		45				65	50	42
		60				72	65	55
		75				81	68	61
		90				90	72	69
UV/H_2_O_2_	---	Time (min)		H_2_O_2_				
				(mL)				
		90		0.2		68	64	58
		--		0.4		76	72	67
		--		0.6		82	76	71
		Time (min)	H_2_O_2_ (mL)		pH			
		90	0.6		3			
		--	--		5	45	35	28
		--	--		7	65	60	40
		--	--		9	68	50	35
						85	65	45
UV/H_2_O_2_/ZnO	---	pH	Time (min)	H_2_O_2_ (mL)	ZnO			
					(g)			
		9	90	0.6	0.3	90	84	82
		--	--	--	0.6	94	87.5	83
		--	--	--	0.9	96	92	90
UV/H_2_O_2_/ TiO_2_	---	pH	Time (min)	H_2_O_2_ (mL)	TiO_2_			
					(g)			
		9	90	0.6	0.3	85	81	79
		--	--	--	0.6	88	84	80
		--	--	--	0.9	94	92	90
UV/H_2_O_2_/SnO_2_	---	pH	Time (min)	H_2_O_2_ (mL)	SnO_2_			
					(g)			
		9	90	0.6	0.3	84	80	78
		--	--	--	0.6	87	82	80
		--	--	--	0.9	92	90	88

The initial concentration of dye is related to the degradation rate. When the initial concentration of dye rises, the path length of photons entering the solution decreases due to more active sites that may be covered with dye ions and a large number of intermediates emerging after the parent dye is degraded, which may interfere with the oxidation process, e.g. reducing the production of ^**·**^OH radical ions, which are important in the photodegradation process, and when the initial concentration of dye falls, the opposite effect occurs^21^. The effects of dye concentration and dye degradation are inversely correlated^22^

### Effect of H2O2

The effect of different doses of H_2_O_2_ ranging from 0.2 to 0.6 mL on the degradation of AV49 was investigated. The degradation efficiency considerably enhanced when H_2_O_2_ was used in the presence of UV radiation. This is due to H_2_O_2_ dissociation in the presence of UV radiations, which produces free hydroxyl radicals, the second most potent oxidizing agent after fluoride^23^. Furthermore, high concentrations of hydrogen peroxide function as scavengers, lowering the concentration of hydroxyl radicals and lowering chemical elimination efficiency^24^.

**Fig. 5:**
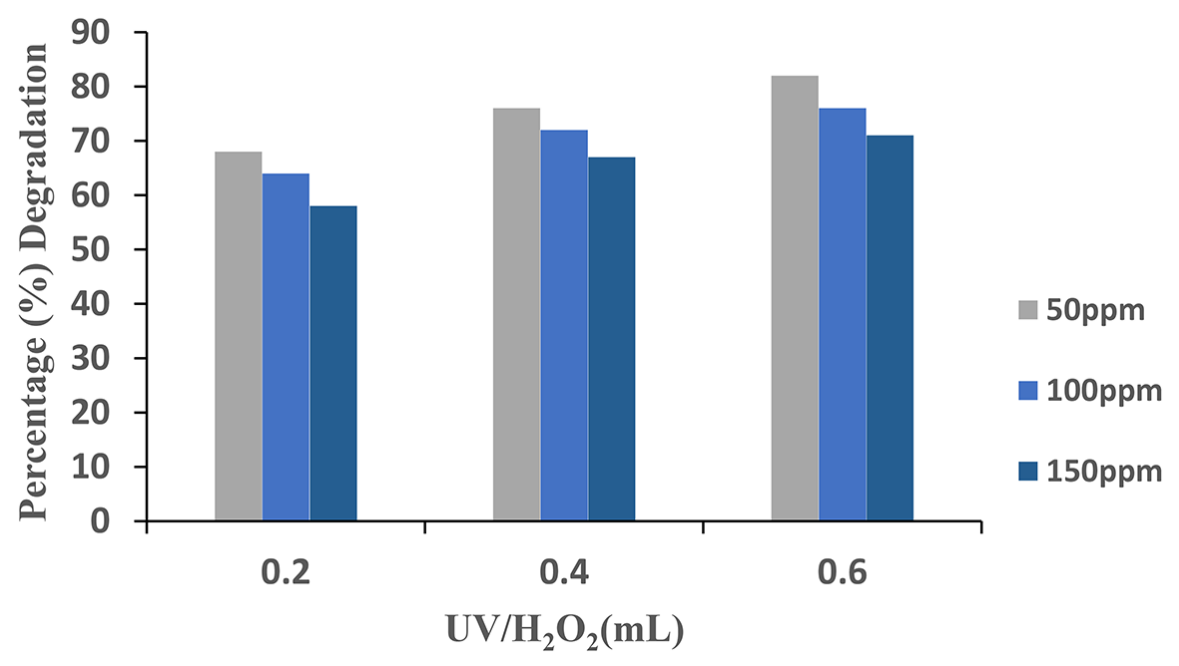
Percentage degradation of AV 49 with various concentrations of H_2_O_2_.

To obtain the best degradation, H_2_O_2_ should be injected at the optimal concentration. It can be examined from (Fig. 5) that by increasing the dosage of H_2_O_2_ ranging from 0.2 to 0.6 mL, the degradation efficiency increased from 68 to 82%. Complete outcomes can be seen in Table 2. Hydroxyl radicals are produced to target AV 49 structure at various points, such as unsaturation spots. AV 49 is transformed to CO_2_ and hetero-atoms in some of these assaults, which are thereafter mineralized. However, in this investigation, the inhibiting effect of a high H_2_O_2_ dose was not seen, most likely because the H_2_O_2_ concentration did not reach such a high level.

### Effect of pH

**Fig. 6:**
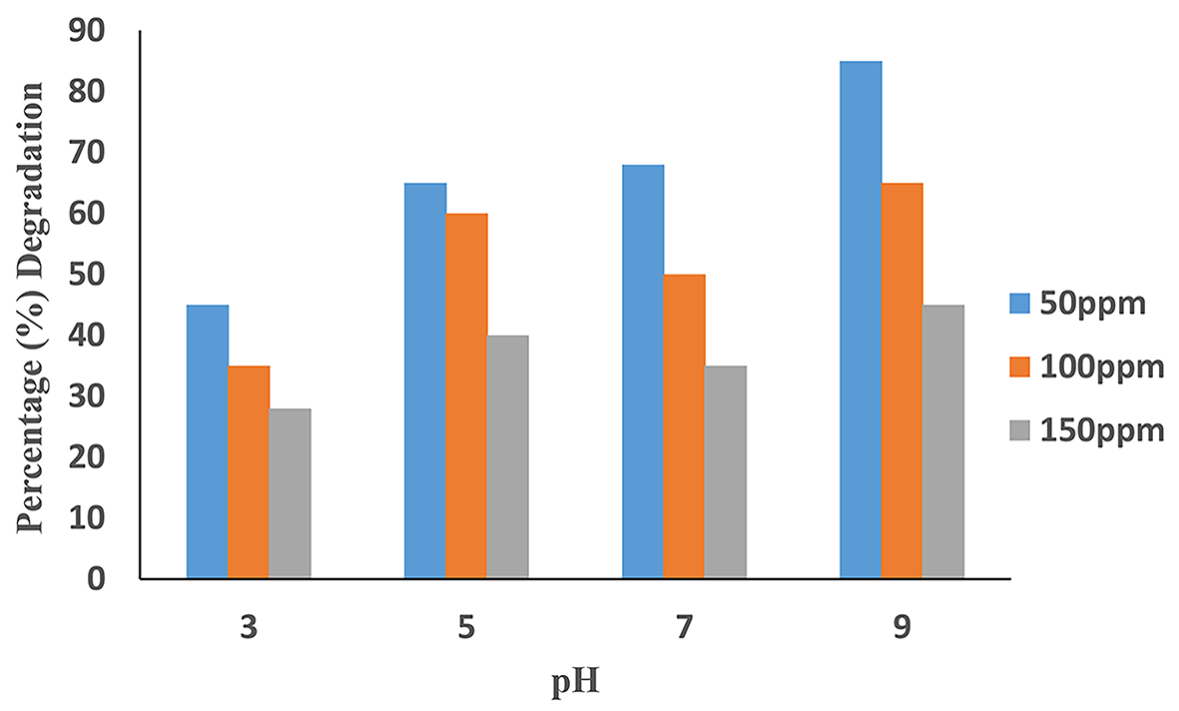
Effect of pH on the degradation efficiency of AV49.

The concentration of AV 49 was studied from the aqueous solution at different pH values. The solution of AV 49 with different concentrations was provided, and HCl and NaOH were used to adjust the solution pH at values of 3, 5, 7, and 9. The results are obtained which show the effect of solution pH on the degradation of AV 49 from the aqueous solution by photodegradation process. (Fig. 6) shows by increasing the initial pH of the solution from 3 to 7, the degradation efficiency was reduced from 80 to 70%. After further increasing the pH from 7 to 9, the degradation efficiency increased from 72 to 96% as shown in (Table 2). That was effectively removed at solution pH 9 and 0.6mL H_2_O_2_. The dye removal was observed to be better in alkaline conditions than in acidic and neutral conditions. Because in an alkaline medium there was an abundance of hydroxyl anions, which involve the photogeneration of hydroxyl radical (^**·**^OH)^25^. Higher concentrations of hydroxyl radical in AV 49 (an anionic dye) result in more efficient photodegradation at higher pH values.

### Effects of UV light-induced photocatalytic degradation

**Fig. 7:**
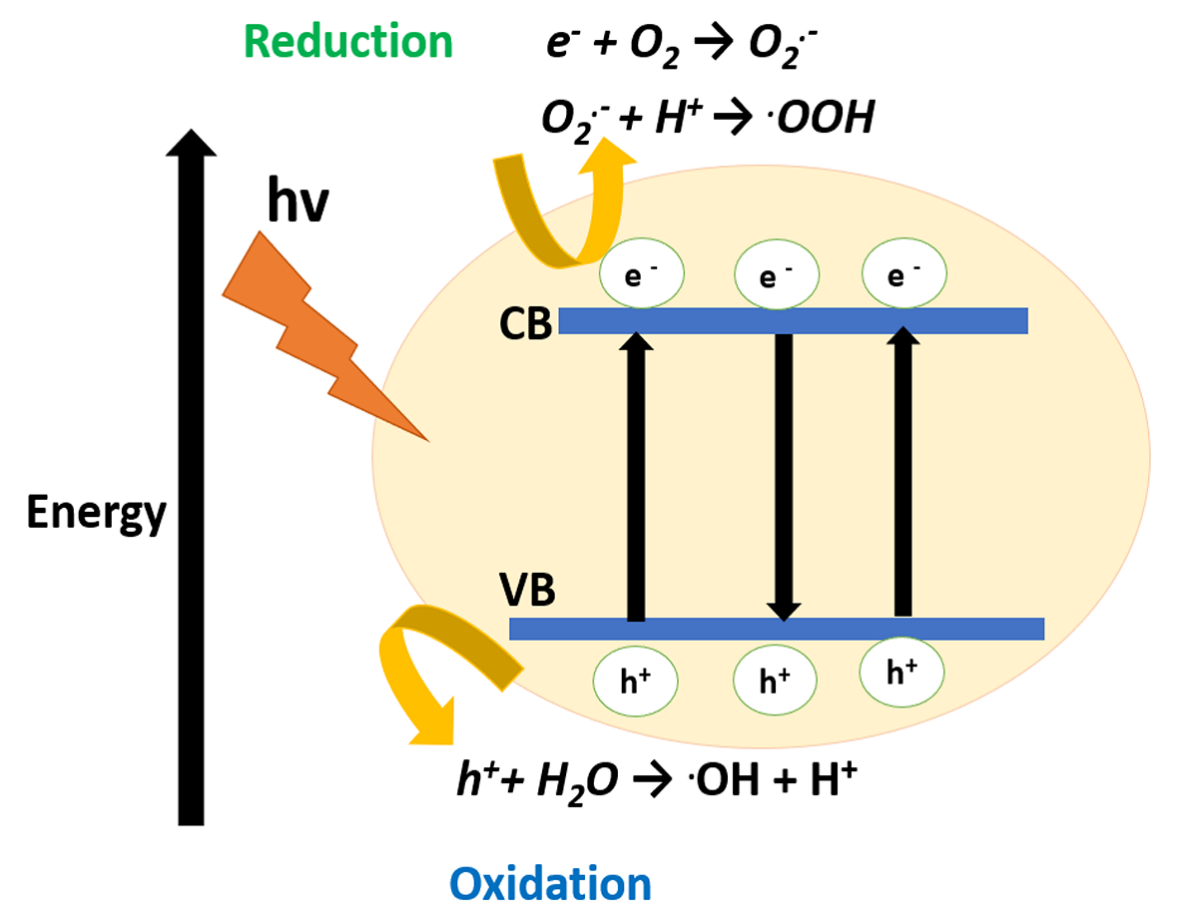
Schematic presentation of photocatalytic degradation of Acid Violet 49.

When UV light is irradiated on the AV49 solution in the presence of the catalyst, electrons are promoted from the valence band to the conduction band, and as a result, a positive hole is produced in the valence band. The electron of the conduction band is then transferred to the surrounding O_2_ and generates O_2_^·−^ as represented in (Fig. 7). As a result, the positive hole reacts with the surface bond water, resulting in the formation of hydroxyl radicals. The breakdown of dye molecules into CO_2_ and water is further aided by these hydroxyl radicals (^**·**^OH)^26^. The effect of catalyst loading on AV49 degradation was examined using TiO_2_, ZnO, and SnO_2_ at concentrations ranging from 0.3 to 0.9 g while maintaining all other parameters constant. Detailed results can be seen in (Table 2).

**Fig. 8:**
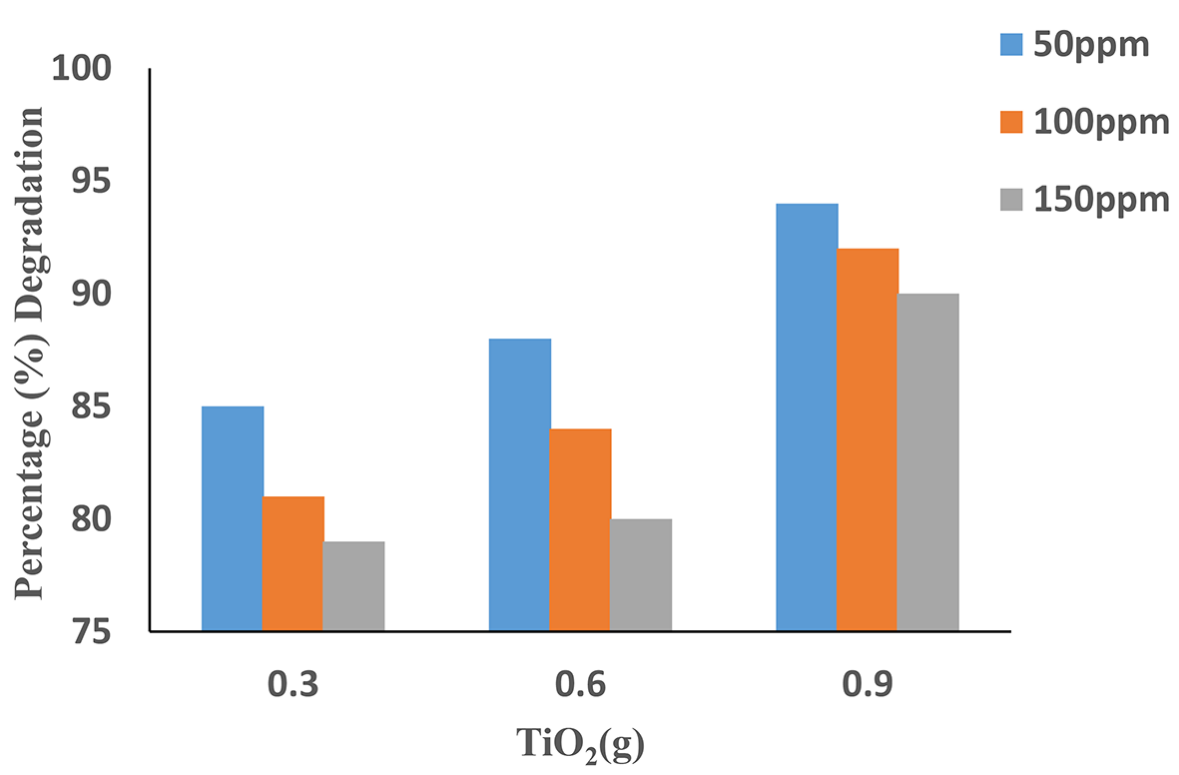
Effects of TiO_2_ on the degradation of AV49. (Experimental conditions: H_2_O_2_ = 0.6 mL, pH = 9, Time = 90 min).

### Effect of TiO2 dosage

The studies were carried out by adjusting catalyst concentrations for the dye solution from 0.3 g, to 0.9 g while keeping all factors constant. The efficiency of degradation increases with the increase of catalytic concentration. Because of the increase in surface area and the availability of more active adsorbent sites. Figure 8 revealed that the rate of degradation reaches its maximum of 94% with 0.9 g of catalyst TiO_2_ at 0.6 mL H_2_O_2_, for 50 mg/L. Excess TiO_2_ and added above the 0.9 g dose did not appreciably improve degradation. Because catalyst particles clumped together, the interfacial area between the reaction fluid and the photocatalyst was reduced. Moreover, it can also increase the opacity of the solution, reducing UV light penetration and hence decreasing degradation efficacy^27^.

### Effect of SnO2

**Fig. 9:**
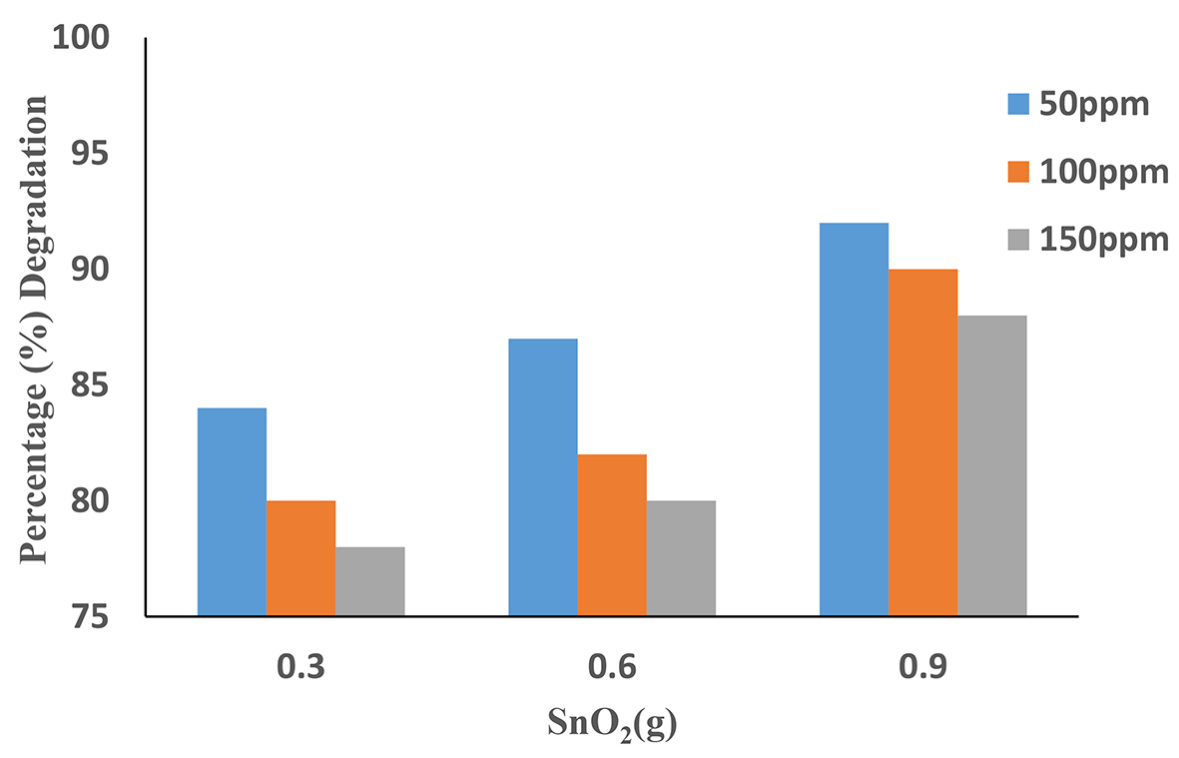
Effects of SnO_2_ dosages on the degradation of Acid Violet 49. (Experimental conditions: H_2_O_2_ = 0.6 mL, pH = 9 and Time = 90 min).

The maximum degradation of AV 49 by SnO_2_ nanoparticles was reported at 92% at a 0.9 g dosage and 0.6 mL H_2_O_2_ for 50 mg/L. As a result, increased SnO_2_ concentration causes a redshift to longer wavelengths. This indicates that with increasing catalytic dosage, the number of accessible active sites for photocatalytic reactions on SnO_2_ nano-catalyst increases thus the generated of free radicals increases^28^. (Fig. 9) shows the maximum degradation of AV49 over SnO_2_ nano-catalyst after 90 min. In the present study (UV/H_2_O_2_/SnO2), because the combination of UV with hydrogen peroxide, the less amount of SnO_2_ was used. At a dose increase from 0.9 g, a slight decrease in reaction time was observed after 30 min, which could lead to high adsorption density because of the overlap of adsorbent sites.

### Effect of ZnO

**Fig. 10:**
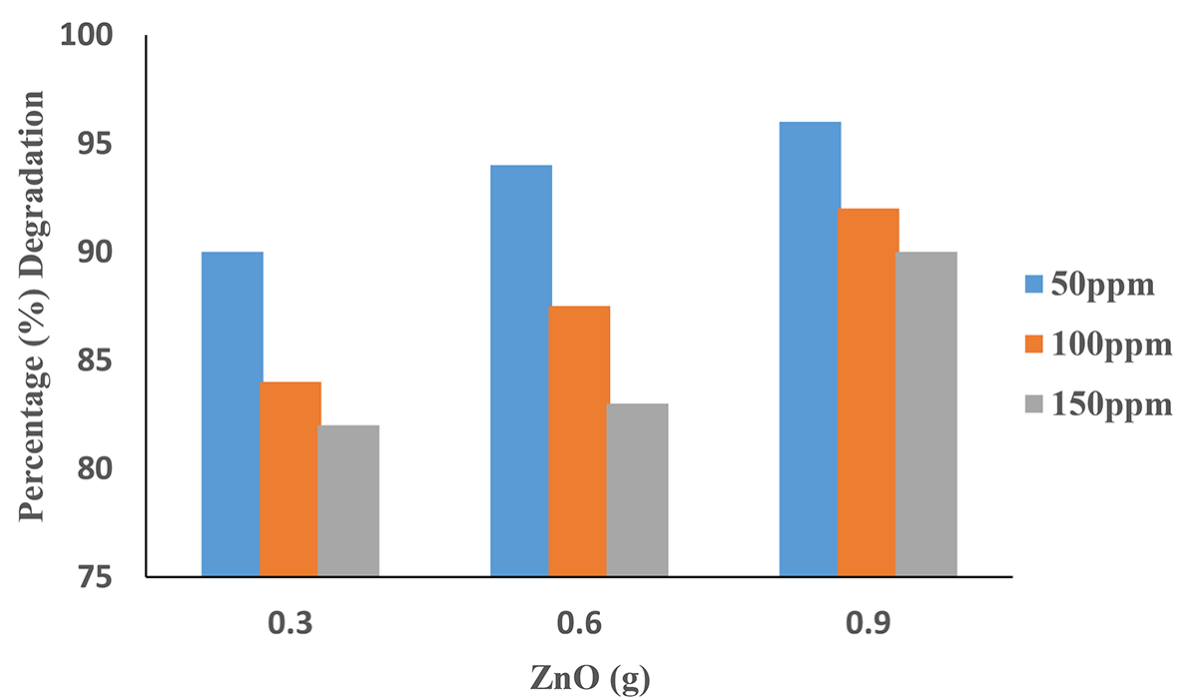
Degradation of dye AV49 at different concentrations of ZnO. (Experimental conditions: H_2_O_2_ = 0.6 mL, pH = 9 and Time = 90 min).

It can be noted from Fig. 10 that the efficiency of degradation increased from 90 to 96% for ZnO with the catalytic dosage ranging from 0.3 to 0.9 g by keeping all parameters constant. The rate of degradation increases with increased catalytic dose due to additional catalytic activating sites and increased electron excitation rate in conjunction with UV, producing more hydroxyl radicals to degrade the dye^29^. Additionally, it is clear from these outcomes that among all of the catalysts, ZnO provided effective percentages of degradation. Then increasing the catalyst dosage from 0.9 g has no noticeable effect on the rate of degradation. It can be explained in terms of a decrease in the penetration of UV light due to the increased effect of scattering and turbidity of the suspension^30^. Moreover, the photo-activated volume of the suspension decreased at the same time. Additionally, reducing the number of active sites due to catalyst agglomeration, makes it difficult to keep the solution homogeneous at a high catalyst dosage^31^.

The TiO_2_ and ZnO catalysts resulted in a higher degree of mineralization of AV49 dye than SnO_2_ in the photocatalytic degradation of AV49. This was ascribed to the supported metal oxides’ improved dispersion compared to SnO_2_, their increased adsorption capacity, and their greater specific surface area of AV 49 supported catalysts. In additon, deposition of ZnO on the AV49 surface prevented the photocorrosion of ZnO that is an important advantage compared to pure ZnO nanoparticles^32^.

### Analysis

#### Toxicological test

Elimination of H_2_O_2_ from irradiated samples was very necessary to avoid its toxic impact. In order to mitigate the effects of H_2_O_2_, small amounts of MnO_2_ (< 1 mg/mL) were added. The solution was clarified and exposed to toxicity tests, including hemolytic, after a specified reaction time of 90 min. The term “infectious disease” refers to hemolysis, which is brought on by microorganisms and other parasites such as Plasmodium falciparum^9^.

**Fig. 11:**
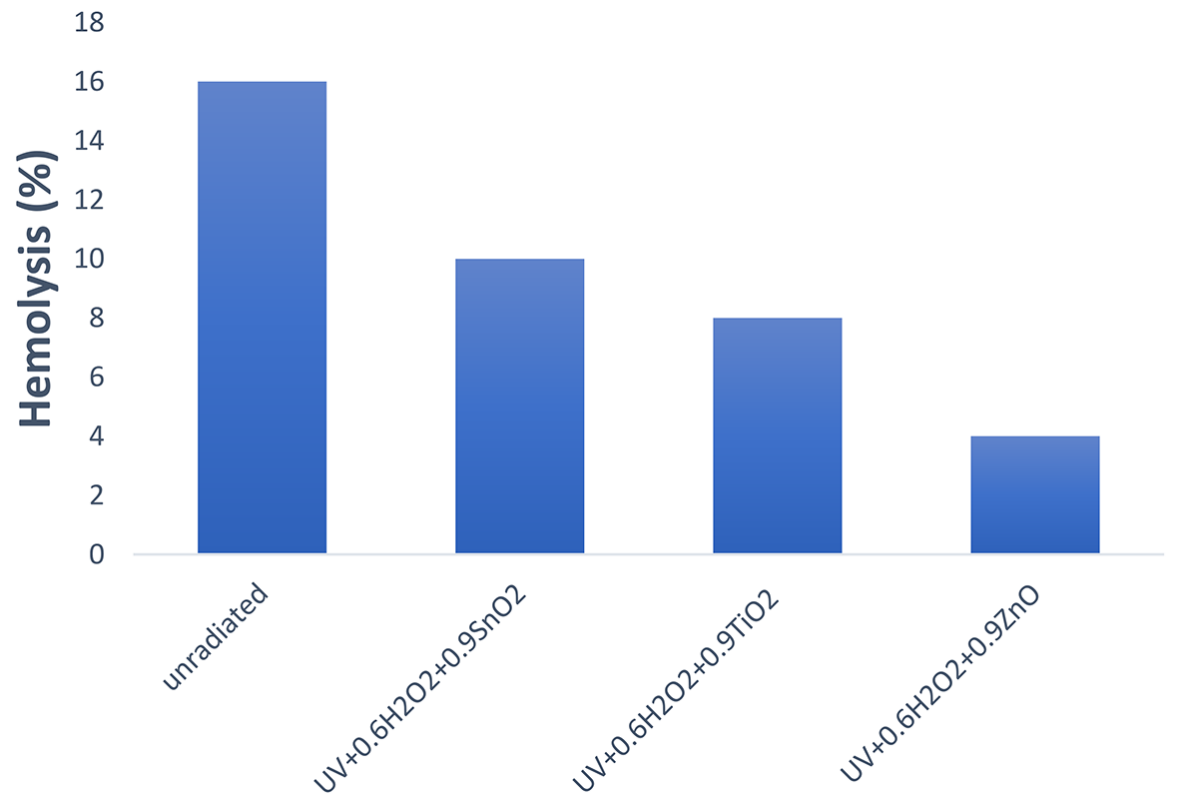
Hemolysis of radiated and unradiated sample of AV49.

The cytotoxicity parameter was observed for untreated and treated samples of AV 49 and results are shown in Fig. 11. The cytotoxicity of un-irradiated 50 ppm was 16% and it was reduced to 10% when irradiated with UV radiation. Further reduction up to 8% and 4% was noticed with the addition of photo-catalyst (ZnO, SnO_2_ and TiO_2_). The Hydrogen peroxide and photo-catalyst ZnO also showed a major effect on the reduction of cytotoxicity of the AV 49.

### FTIR Analysis

**Fig. 12:**
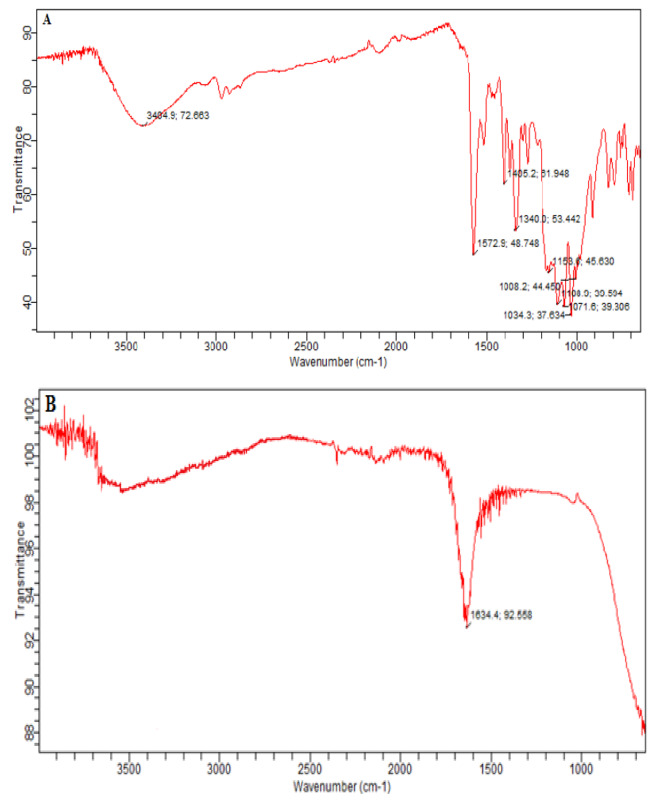
FTIR spectrum of AV49 (**A**) before radiated and (**B**) after radiated sample (UV/ZnO/H_2_O_2_: Time = 90 min, [H_2_O_2_] = 0.6 mL, [ZnO] = 0.9 g, pH = 9, [AV49] = 50 mg/L)

FT-IR spectroscopy is an active, sensitive and non-destructive physical technique for the examination of organic compounds^32^. Both the treated and untreated Acid Violet 49 molecules were subjected to FT-IR spectroscopy in order to analyze the functional groups and covalent bonds within the molecules. (Fig. 12A) revealed that the peak at 1158 cm^− 1^ in the AV49 spectrum indicates an asymmetric -S = O stretching in the -SO_3_Na group, while 1,340.07 cm^− 1^ shows the C-N stretching vibration in aromatic amines. Furthermore, the faint peak at 1,405.2 cm^− 1^ shows the stretching vibration of C = C in the aromatic ring, while the highlighted strong peak at 1572.9 cm^− 1^ indicates the C = N stretching. The degradation spectra of the AV 49 dye following UV/H_2_O_2_/ZnO treatment are shown in (Fig. 12B), where it is evident that the aromatic ring was destructed and the pre-treatment peaks at 1,340.07 cm^− 1^, 1158 cm^− 1^, and consecutive peaks at 1572.9 cm^− 1^ and 1,405.2 cm^− 1^ disappeared.

### LC/MS analysis

LC-MS is the most prominent analytical technique used for identifying the degraded products as well as to explain the chemical structure. Furthermore, Electrospray Ionization methods in mass spectroscopy can offer information regarding the mass of degraded products under investigation^33^.

**Fig. 13:**
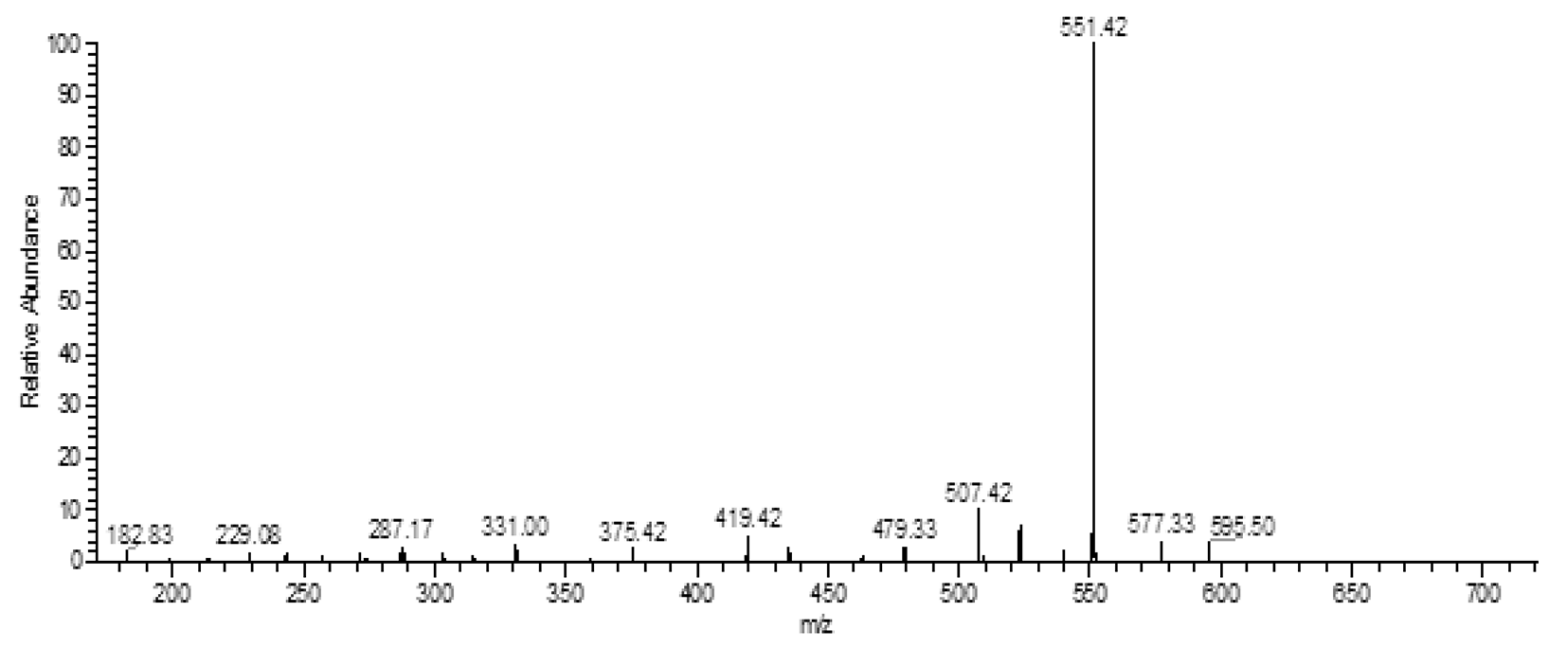
LCMS spectra after treatment of Acid Violet 49 sample (UV/ZnO/H_2_O_2_: Time = 90 min, [H_2_O_2_] = 0.6 mL, [ZnO] = 0.9 g, pH = 9, and [AV49] = 50 mg/L).

**Fig. 14:**
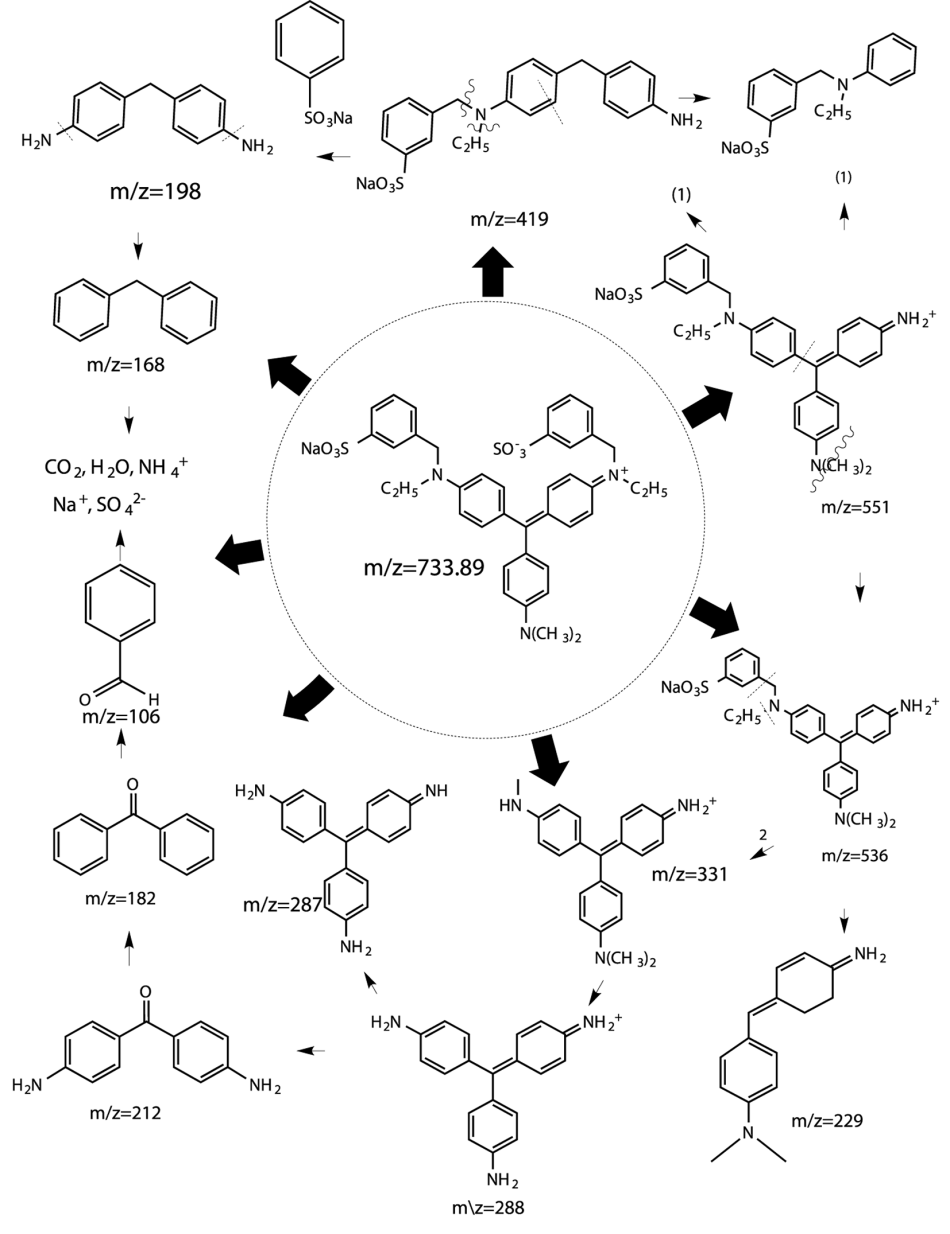
Plausible degradation pathway of Acid Violet 49 investigated by LC-MS.

LC-MS analyses were performed using a Linear ion trap mass spectrometer in positive mode with electrospray ionization for evaluating the degradation product of AV 49 by interpreting their mass spectra data, which displayed their molecule ion peaks concerning m/z value. The degraded product ion with a mass to charge ratio (m/z) was scanned from 160 to 900, the retention time was 5.58 min, and base peak intensity was 4.86 × 10^1. (Fig. 13) shows the LC-MS spectrum of AV 49 after treatment with UV/H_2_O_2_/ZnO and the degraded pathway of AV 49 is proposed in (Fig. 14). Acid Violet 49 belongs to the triarylmethane class of dyes. The main species involved in the degradation process is hydroxyl radical (.OH) which is generated by the combination of ZnO and H_2_O_2_, attacks the (C = C, C-N bond) near the chromophore (Para-quinoid group), and the C-S bond between the aromatic ring and the sulfonate group and lead to the formation of different degraded product with m/z value at 182.83, 287.17, 229.08, 331.00, 507.42, and 419.42. The fading of the colors is due to the breakage of the C-N and C = C bonds. Possibly the configured by-products are shown in (Table 3).

**Table 3:** Degradation products of Acid Violet 49 identified by LC-MS.

No	Compound Name (Formula)	Structural Formula	m/z	Status
1	Benzophenone (C_13_H_10_O)		182.83	Detected
2	Pararosaniline (C_19_H_17_N_3_)		287.17	Detected
3	4-((4-(dimethylamino)phenyl (4-(methylamino)phenyl) methylene)cyclohex-2,5-dieniminium (C_12_H_34_N_3_^+^)		331.00	Detected
4	Sodium3-(((4-benzylphenyl) amino)methyl)benzenesulfonate (C_20_H_18_NNaO_3_S)		375.42	Detected
5	Sodium-3-{((4-(4- aminobenzyl)phenyl)(ethyl) methyl)benzenesulphonate (C_22_H_23_N_2_NaO_3_S)		419.42	Detected

## Conclusions

The findings of this study demonstrate the effectiveness of UV, UV/H_2_O_2_, UV/H_2_O_2_/ZnO, UV/H_2_O_2_/SnO_2_, and UV/H_2_O_2_/TiO_2_-based processes on the degradation of Acid Violet 49. The experimental findings show that increasing the concentration of AV49 decreases the efficiency of degradation while increasing the pH increases efficiency. Moreover, increasing concentrations of nanoparticles and hydrogen peroxide led to increasing the percentage of degradation. Under optimum conditions, complete degradation of AV 49 (initial concentration 50ppm, 0.6mL H_2_O_2_, 0.9 g ZnO, and pH = 9) was achieved in 90 min. In the case of UV irradiation, 82% degradation of AV49 was achieved by using the UV/H_2_O_2_ process. Effective degradation of 92%, 94%, and 96% was achieved under optimum conditions by using UV/H_2_O_2_/ZnO, UV/H_2_O_2_/SnO_2_, and UV/H_2_O_2_/TiO_2_ based processes. The degradation rate in the basic solution was higher than in the acidic solution. FTIR spectrum before and after the treatment revealed the disappearance of the function group. LC-MS analysis revealed the degradation of Acid Violet 49 into intermediates. The reaction pathway was proposed as the hydroxyl radical attack and removed the substituents on the side chain of Acid Violet 49. Consequently, the photocatalytic process proved to be efficient in the degradation of AV49 dye in an aqueous solution.

## Data Availability

All data generated or analyzed during this study are included in this published article.
